# Observations on Certain of the Diseases of Young Children

**Published:** 1850-11

**Authors:** 


### BIBLIOGRAPHICAL NOTICES.

*Observations on certain of the Diseases of Young Children.* By CHARLES D. MEIGS, M. D., &c. &c. 8vo. Philadelphia, Lea & Blanchard.

